# Novel Lutein Loaded Lipid Nanoparticles on Porcine Corneal Distribution

**DOI:** 10.1155/2014/304694

**Published:** 2014-07-02

**Authors:** Chi-Hsien Liu, Hao-Che Chiu, Wei-Chi Wu, Soubhagya Laxmi Sahoo, Ching-Yun Hsu

**Affiliations:** ^1^Graduate Institute of Biochemical and Biomedical Engineering, Chang Gung University, 259 Wen-Hwa First Road, Kwei-Shan, Tao-Yuan 33302, Taiwan; ^2^Research Center for Industry of Human Ecology, Chang Gung University of Science and Technology, 261 Wen-Hwa First Road, Kwei-Shan, Tao-Yuan 33303, Taiwan; ^3^College of Medicine, Chang Gung University, 259 Wen-Hwa First Road, Kwei-Shan, Tao-Yuan 33302, Taiwan; ^4^Department of Ophthalmology, Chang Gung Memorial Hospital, 5 Fusing Street, Kwei-Shan, Tao-Yuan 33305, Taiwan

## Abstract

Topical delivery has the advantages including being user friendly and cost effective. Development of topical delivery carriers for lutein is becoming an important issue for the ocular drug delivery. Quantification of the partition coefficient of drug in the ocular tissue is the first step for the evaluation of delivery efficacy. The objectives of this study were to evaluate the effects of lipid nanoparticles and cyclodextrin (CD) on the corneal lutein accumulation and to measure the partition coefficients in the porcine cornea. Lipid nanoparticles combined with 2% HP*β*CD could enhance lutein accumulation up to 209.2 ± 18 (*μ*g/g) which is 4.9-fold higher than that of the nanoparticles. CD combined nanoparticles have 68% of drug loading efficiency and lower cytotoxicity in the bovine cornea cells. From the confocal images, this improvement is due to the increased partitioning of lutein to the corneal epithelium by CD in the lipid nanoparticles. The novel lipid nanoparticles could not only improve the stability and entrapment efficacy of lutein but also enhance the lutein accumulation and partition in the cornea. Additionally the corneal accumulation of lutein was further enhanced by increasing the lutein payload in the vehicles.

## 1. Introduction

Carotenoids act as quenchers of oxygen free radicals, blockers of blue-light damage, and inhibitors of lipid peroxidation [1]. Intense exposure to ultraviolet radiation and high oxygen tension render the cornea particularly vulnerable to oxidative damage. The role of oxidative stress has been studied in the corneal diseases including keratitis, keratoconus, infection, and inflammation [2]. Antioxidants such as tocopherol and epigallocatechin gallate can protect cornea from free radicals in the animal model [3, 4]. Lutein is reported to suppress the development of endotoxin-induced uveitis in the rat model by inhibiting the NF-*κ*B dependent signaling pathway and the subsequent production of proinflammatory mediators [5]. Lutein also has neuroprotective effects against the neural damage of retina caused by inflammation [6]. The pathogenic mechanism of macular degeneration and the protective role of lutein have been extensively investigated [7, 8]. However, ocular lutein delivery remains challenging because of the isolated structure of eye. The conventional eye drops are restricted to deliver lipophilic drug due to the existence of the anatomical barriers such as cornea and sclera [9]. Occasionally, intravitreal injections cause side effects such as endophthalmitis, cataract formation, and retinal detachment [10]. There is a need to develop novel drug delivery carriers capable of increasing ocular bioavailability and decreasing the side effects. Nanoparticles may overcome the physiological barriers and deliver the drug to the target by virtue of their nanoscale and functionalization [11]. Among several nanoparticles, lipid nanoparticles such as nanoscaled lipid carriers (NLCs) have been recognized as an interesting and promising topical delivery vehicle for lipophilic drugs [12]. NLCs are especially useful in ocular drug delivery as they can enhance the ocular retention and corneal absorption and can also improve the ocular bioavailability [13]. These enhancing mechanisms include the adhesiveness of nanoparticles to the cornea and penetration by lipids on the epithelial layer. The lipids in NLCs have other advantages like biocompatibility, high drug payload, and degradation protection.

Cyclodextrins (CDs) contain several glucose units that form a hydrophobic central cavity for lipophilic drugs and a hydrophilic outer surface for water solubility [14]. CDs are functional additives in the formulations to enhance drug stability and to decrease drug irritation [15]. CDs also act as a permeation enhancer at the cornea by increasing the drug retention at the surface of the corneal epithelium [16]. Hydroxyalkylation of *β*CD can increase the water solubility of parent *β*CD by converting *β*CD into its amorphous and noncrystallizable derivatives and can also reduce hemolytic and renal toxicities of parent CDs [17]. Modified lipid nanoparticles not only can modulate the biodistribution of the loaded drug but also control the absorption rate of drugs administered [18]. For example, solid lipid nanoparticles of paclitaxel (PTX) modified with HP*β*CD can enhance cellular accumulation of the drug into p-glycoprotein expressing cells [19]. The cysteine-polyethylene glycol stearate modified nanostructured lipid carrier has the mucoadhesive properties on the surface of rabbit eyes. The encapsulated cyclosporine in the hybrid carriers can remain on the ocular surface nearly up to 6 hours [20]. The coating can improve drug absorption due to its ocular adhesiveness or permeability enhancer properties. These results suggest modified nanoparticles as promising vehicles for ocular delivery.

Since the cornea is one of the main barriers for the ocular transport, therefore the distribution of lutein in the cornea is important for the evaluation of topical delivery efficacy. Besides this limited literature data for corneal partition coefficient are available for lutein. Our primary objectives were to evaluate the distribution profile of lutein in the porcine cornea and to study the formulation effects on the corneal partition coefficient of lutein by using NLCs as the vehicles. The porcine cornea was established as an in vitro model for the characterization of lutein accumulation and distribution. Also the novel NLCs were analyzed for their morphology, lutein encapsulation efficacy, and cytotoxicity.

## 2. Materials and Methods

### 2.1. Reagents and Chemicals

The lutein (Lutemax) is kindly provided by DKSH (Taipei, Taiwan). Cyclodextrins are purchased from Wako Pure Chemical Industries (Osaka, Japan). Transcutol HP is obtained from Gattefossé (Lyon, France). Tween 80 and Span 60 are supplied by Kanto Chemical (Tokyo, Japan). All other chemicals and reagents are purchased from Sigma-Aldrich unless otherwise stated.

### 2.2. Preparation of Lutein-Loaded NLC

Lutein-loaded NLCs and lutein-loaded NLCs combined with CD were manufactured by a hot sonication method. Briefly, lutein was first dissolved in diethylene glycol monoethyl ether (Transcutol) at the concentration of 2%. Lutein in Transcutol (1.2 g) was mixed with decanoic acid (60 mg) and Span 60 (120 mg) at 80°C in a dry bath. Tween 80 (180 mg) and CD (30~180 mg) were separately dispersed in 4.44 mL of distilled water. Finally, the mixture solution was sonicated for 5 min using a probe-type sonicator. Samples were stored in the dark for further characterization. Bare NLC and NLC-CD were made by the same method with no lutein addition. Composition of the developed vehicles including NLC and NLC + HP*β*CD is shown in Table 3.

### 2.3. Characterization of NLC

The size and surface property of MNP were characterized by TEM. A drop of diluted sample was dispersed onto a 100-mesh copper grid, and then the excess drop was removed with a filter paper. The sample containing copper grid (CF200-Cu, Electron Microscopy Science, Washington, DC) dried for two hours at 55°C prior to TEM analysis. The morphology of the various MNP was observed by TEM (JEOL JEM 2000 EXII, Tokyo, Japan). NLC samples were diluted 1 : 25 with Milli-Q water and dried on carbon film (CF200-Cu, Electron Microscopy Science, Washington, PA, USA) for 12 hours. After being stained with a 1% solution of phosphotungstic acid (Merck, Darmstadt, Germany) for 30 seconds and vacuum-dried in the incubator, samples were then analyzed by TEM. The average particle size and zeta potential in different formulations were characterized by using Zetasizer Nano ZS 90 (Malvern, Worcestershire, UK) at a fixed angle of 90° and a temperature of 25°C. The smaller the PI, the more uniform the size distribution of dispersion. Zeta potential characterizes the surface charge of particles, which is an indicator of the long-term stability. Zeta potential values of ±30 mV and above represent a stable formulation. Samples were diluted with water to a suitable concentration before the analysis of size distribution.

### 2.4. Estimation of Lutein Partition Coefficient

To investigate possible drug penetration or retention in the cornea, the following experiments were performed to measure partition coefficient and accumulation rate of lutein in the cornea or sclera. The tendency of lutein into scleral tissue was estimated by measuring its partition coefficient between porcine sclera and our formulation at the temperature of 32°C. Lutein dissolved in the vehicle for the partition experiments at a concentration of 2000 *μ*g/mg. The vehicle (2 g) was added to a previously weighed amount of porcine (sclera (18–22 mg), cornea (25–30 mg)) in a 10 mL vial. The lutein vehicle and the ocular tissues were incubated at the shaker with 200 rpm agitation for 4 h. Three scleral tissues were randomly withdrawn from the vial, rinsed with phosphate buffer saline, wiped with paper, and weighed. The ocular tissue was then homogenized and the lutein accumulated in tissue was extracted by using 2 mL of the extraction buffer (10% tetrahydrofuran and 90% methanol). The homogenized solution was centrifuged at 10000 rpm, filtered by a nylon filter with 0.22 *μ*m pore size, and analyzed by HPLC to determine total lutein content (*W*_*e*_, *μ*g) in the extraction buffer. Each experiment was replicated at least four times. The drug concentration [*C*_*c*_] in the cornea after the incubation period was calculated as previously described with some modification [21]:

(1) Cc=WdWc,

where *W*_*d*_ is the lutein amount in the cornea tissue and *W*_*c*_ is the weight of the cornea.

Finally, the partition coefficient (*K*) was then calculated using the formula:

(2) $$\begin{array}{c} K\;=\frac{C_{c}}{C_{i}}, \end{array}$$

where [*C*_*i*_] is the initial concentration of lutein in the vehicle.

### 2.5. Estimation of Lutein Entrapment Efficiency

The prepared nanoparticles were separated from the free lutein using a Sephadex G-50 (GE Healthcare) resin for measurement of entrapment efficiency. The lutein-loaded vehicle of 0.1 mL was separated by using 2 mL of resin. The part of the outflow with opalescence and metered volume to 6 mL were collected and measured by HPLC. The entrapment efficiency (EE) of lutein in the nanoparticles was calculated according to the following equations [22]:

(3) $$\begin{array}{c} \text{EE}=\frac{W_{e}}{W_{i}}\times 100\%, \end{array}$$

where *W*_*i*_ represents the initially added amount of drug and *W*_*e*_ drug represents the amount of drug entrapped in the nanoparticles. The HPLC system (Jasco, Tokyo, Japan) consists of a pump, a UV detector, and a Microsorb-C18 column (Varian, Lake Forest, CA, USA). The mobile phase was composed by 10% (v/v) tetrahydrofuran and 90% (v/v) methanol. The flow rate was 1.0 mL/min and the effluent was monitored at 450 nm as previously described [23]. The lutein is well separated at the retention time 3.85 minutes.

### 2.6. Preparation of the Ocular Tissues and Histological Examination

Porcine eyes are kindly donated by Ya-Hsen Frozen Foods (Taoyuan, Taiwan). The eye balls are freshly collected and stored in ice during transport. The cornea is dissected within 24 hours of slaughter, wrapped in wetted tissue paper, and stored at −80°C in a polypropylene bag and used within one month [24]. Porcine ocular samples were treated with PBS and CD formulation, respectively, for 24 h on a Franz diffusion cell. Thereafter, ocular tissue was fixed in PBS solution containing 10% formalin and cut vertically, dehydrated using ethanol, embedded in paraffin, and stained with hematoxylin and eosin (H&E) staining. These samples were then observed under light microscope (Olympus BX51, Tokyo, Japan) using 100 magnification. For penetration analysis by confocal laser scanning microscopy, the ocular tissue was removed from the lutein solution and rinsed with PBS and then the surface of the sclera was wiped gently. The ocular tissue was directly sandwiched between a glass slide and a cover slip and examined by using confocal microscopy without additional tissue processing. We used a Leica TCS SP2 confocal laser scanning microscope (Leica, Heerbrugg, Switzerland) to assay the lutein corneal distribution. Lutein fluorescence emitted at 515 nm was recorded when excited at a wavelength of 488 nm by means of an argon laser. The sample was scanned from the tissue surface (0 *μ*m) to a depth of 352 *μ*m at a 29.3 *μ*m interval.

### 2.7. Cell Culture and Cytotoxicity of Vehicles

Bovine cornea endothelia (BCE, type: C/D-1b) cells were purchased from the BCRC (Hsinchu, Taiwan). BCE cells were cultured in Dulbecco's modified Eagle's medium (DMEM) supplemented with 10% fetal bovine serum (FBS) in a humidified atmosphere of 5% CO_2_ at 37°C. Cells were subcultured using trypsin-EDTA when reaching 80% confluence. For cytotoxicity experiments, BCE cells were seeded on 48-well plates at a density of 5 × 10^4^ cells/well and allowed to grow for 48 hours. NLCs were diluted to a series of concentrations using DMEM medium. Cells were fixed by 0.2 mL of 4% formaldehyde and the nucleus stained by Hoechst 33342 and then analyzed by INCell Analyzer 1000 (GE Healthcare, Piscataway, NJ) at the end of the incubation. This high throughput analyzer is designed for cellular imaging assays. The fluorescence at 455 nm in the nucleus stained by Hoechst 33342 was recorded after excitation at 350 nm. The cell viability is defined as follows: (the number of nuclei in treated cells/ the number of nuclei in controlled cells) × 100%.

### 2.8. Statistical Analysis

Statistical analysis of differences between different treatments was performed using the Student's *t*-test. A 0.05 level of probability was taken as the level of significance. An analysis of variance (ANOVA) test was also used.

## 3. Results and Discussion

### 3.1. NLC Composition on Corneal Lutein Accumulation

**Figure 1:**
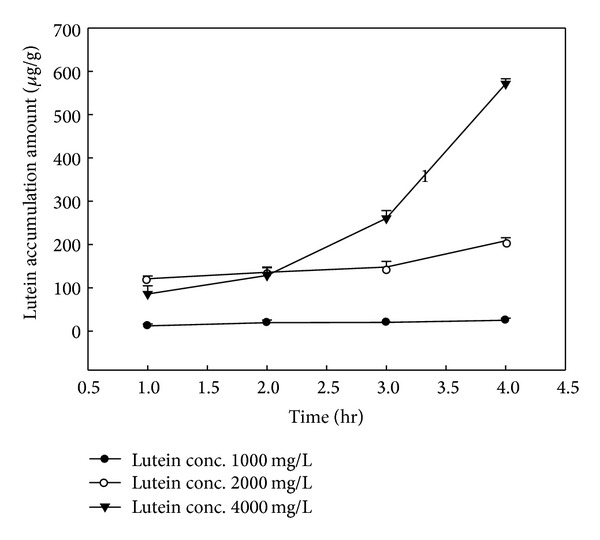
Dose effect of lutein on corneal accumulation using 2% HP*β*CD combined NLCs.

In order to evaluate the corneal lutein accumulation by CD combined NLC, the accumulation of lutein in the cornea was examined using the porcine cornea. The accumulation rate and partition coefficient of drug in targeting tissue are important for the evaluation of delivery efficacy. We investigated the effects of combination of various CDs with NLCs on lutein accumulation in the corneal tissue during the four-hour period. As shown in Table 1, the corneal accumulation and partition coefficient of lutein in combination with NLCs increase in the order of *α*-CD < *β*-CD < He*β*CD < HP*β*CD. The stimulatory effect of the CD in NLCs for lutein accumulation in the cornea was confirmed. The partition coefficient between the porcine cornea and the vehicles was calculated by assuming the tissue to be homogenous and the diffusion into cornea to be passive. Since the lutein accumulation increased in the same order, therefore similar trend was observed for the partition coefficient of lutein in the porcine cornea. The partition coefficient of NLC + HP*β*CD was significantly higher (*P* < 0.05) than that of NLC, whereas no significant difference (*P* > 0.05) existed between the two vehicles NLC + *α*CD and NLC + *β*CD. The average lutein accumulation rates in cornea for NLC + 0.5% HP*β*CD and NLC were 19.8 ± 0.2 and 10.6 ± 0.4 *μ*g g^−1^ hr^−1^, respectively. NLC combined with 0.5% HP*β*CD could elevate the lutein accumulation 1.8-fold as compared to that of NLC alone. Besides, the addition of CD in the NLC at the concentration of 0.5% did not affect the stability of formulation. CD-based formulations have successfully delivered various drugs into the eye [15, 25]. Among the tested CDs, HP*β*CD effectively enhanced the lutein accumulation in the porcine cornea. We consequently investigated the effect of amount of HP*β*CD in NLC carriers on the lutein accumulation. The dosages of HP*β*CD in NLC on the corneal lutein accumulation are shown in Table 2. The concentration of HP*β*CD in the NLC up to 2% could enhance the lutein accumulation in the porcine cornea. No further increase in the lutein accumulation is observed for more than 3% HP*β*CD in the NLCs. The instability of NLC + 3% HP*β*CD might account for reducing accumulation since the phase separation was observed in this vehicle. Among the tested HP*β*CD concentration, 2% HP*β*CD in NLC carrier could significantly enhance the lutein accumulation in the porcine cornea. The average lutein accumulation rate in cornea for NLC + HP*β*CD is found to be 52.2 ± 1.8 *μ*g g^−1^ hr^−1^, indicating the lutein accumulation to be 4.91-fold as compared to that of NLC alone. The payload effect of lutein (1000~4000 *μ*g g^−1^) in the NLC + HP*β*CD vehicle on corneal lutein accumulation is depicted in Figure 1. Enhancing effect of the lutein concentration on corneal accumulation was observed using the optimal formulation. The amount of lutein permeating into the pig cornea gradually increased when lutein in the vehicles increased from 1000 to 4000 *μ*g g^−1^. The elevation of the lutein load in the vehicles could improve the driving force needed for the diffusion of lutein into the cornea. Fick's first law postulates that the diffusion flux goes from regions of high concentration to regions of low concentration, with a magnitude that is proportional to the concentration gradient [26]. As shown in Table 2, the partition coefficient and the accumulation rate of NLC + HP*β*CD (4000 *μ*g g^−1^ lutein) were significantly higher (*P* < 0.05) than those of NLC + HP*β*CD with 2000 *μ*g g^−1^ lutein. Similarly, the lutein accumulation rates in cornea for 4000 and 2000 *μ*g g^−1^ lutein in NLC + HP*β*CD were 142.8 ± 3 and 52.2 ± 1.8 *μ*g g^−1^ hr^−1^, respectively. The increase in lutein concentration also stimulated the partition coefficient in the porcine cornea. NLC + HP*β*CD loaded with 4000 *μ*g g^−1^ lutein could elevate the lutein accumulation 13-fold as compared to that of NLC + HP*β*CD loaded with 2000 *μ*g g^−1^ lutein. Phase separation was induced after 3 weeks storage in the cargo of NLC + HP*β*CD with 4000 *μ*g g^−1^ lutein. The payload effect of 2000 *μ*g g^−1^ lutein in the NLC + HP*β*CD vehicle was investigated in the following sections.

### 3.2. Corneal Lutein Delivery and NLCs Characterization

**Figure 2:**
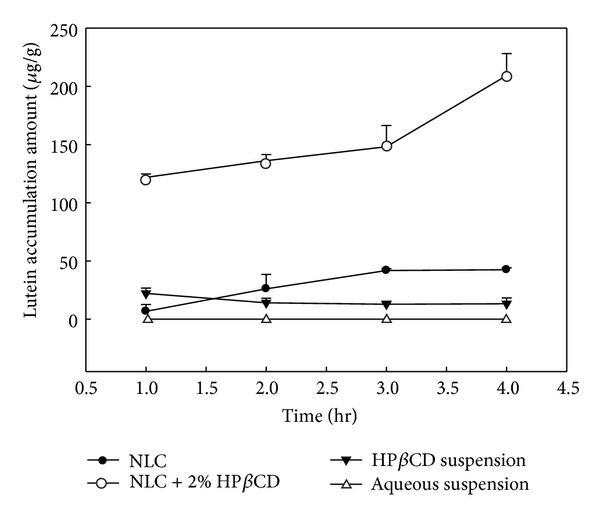
Effect of vehicles on lutein corneal accumulation. The lutein content in all vehicles is 2000 *μ*g/g.

**Figure 3:**
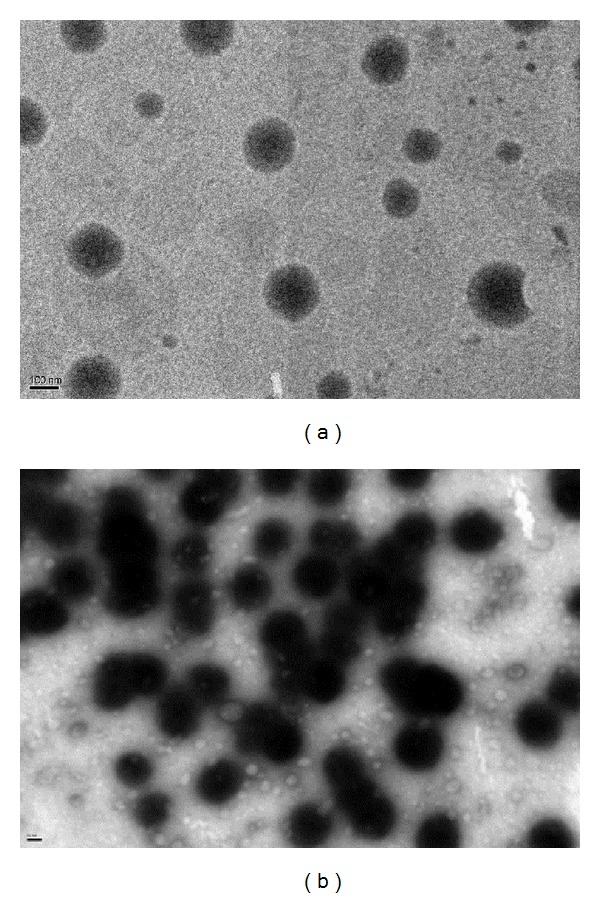
TEM photographs for NLC(A) and NLC + 2% HP*β*CD (100000x). Scale bar in (a) and (b) represents 100 and 50 nm, respectively. The size of the NLCs was 190 and 360 nm, respectively.

In order to understand the mechanism of the enhancing effect of HP*β*CD, the characterization of the four vehicles and lutein distribution pattern in a porcine cornea were performed. The compositions of the four vehicles including NLC, NLC + HP*β*CD, HP*β*CD suspension, and aqueous suspension are shown in Table 3. Capric acid, a natural fatty acid, was used as a solid lipid in the NLC. Corn oil and the liquid oil were mixed with capric acid into which the lutein was incorporated. Corn oil was used as a carrier for lutein in the DKSH product. Our NLC system was stabilized by using the two surfactants Tween 80 (with a hydrophile-lipophile balance (HLB) of 15) and Span 60 (with an HLB of 4.7). Combinations of hydrophilic and lipophilic emulsifiers have been proved to maintain a stable dispersion by imparting more rigidity and strength to the binary-surfactant film [27]. In addition, surfactants can reduce the colloidal size by reducing the surface tension and fluidizing the interfacial droplet film. The HLB of surfactant blends (Tween 80/Span 60) were found to be 10.9 by multiplying the weight percentages of surfactants and their individual HLB values. We observed that the droplet sizes in the surfactant-free suspensions were significantly larger than those of the NLC and NLC + HP*β*CD. Phase separation in the aqueous suspensions was observed after a short storage. The vehicle effect on corneal lutein accumulation is demonstrated in Figure 2. The lutein-loaded vehicles including NLC, NLC + HP*β*CD, HP*β*CD suspension and aqueous suspension had significant difference in corneal lutein accumulation. Lutein droplets were formed when lutein was suspended in the water and almost no lutein accumulation in the porcine cornea was observed. The lutein suspended in the HP*β*CD alone could hardly enhance the lutein accumulation in the cornea. The limited contact area between the big droplets and the cornea contributed to the low tissue accumulation. The partition coefficient of lutein in vehicle of NLC + HP*β*CD was about 15-fold higher than that of HP*β*CD suspension. This result clearly indicates the important role of NLCs in the enhancing effect of corneal accumulation. Only HP*β*CD could not efficiently enhance the corneal accumulation of lutein. As demonstrated in Table 3, the zeta potentials of these vehicles lie in the range −28 to −38 mV. The addition of CD into NLCs would decrease the zeta potential on the surface of NLC. The size distribution of the NLCs is smaller than that of NLC + HP*β*CD. TEM photographs provided the information for the NLCs microstructure. HP*β*CD (white aggregation) around the NLCs was observed in the vehicle of NLC + HP*β*CD as shown in the TEM image. The adhesion of CDs on the surface of NLCs resulted in the increase in particle size. The spherical morphology of lutein-loaded NLCs and NLC + HP*β*CD could be clearly observed using TEM. From the TEM image (Figures 3(a) and 3(b)), the mean sizes of the NLCs and NLC + HP*β*CD are found to be 190 and 360 nm, respectively, which is consistent with the size measured by photon correlation spectroscopy (Table 3). The location of the particles is randomly distributed around the NLCs (Figure 3(b)). There are two reasons that may contribute to the existence of the nanoparticles. Firstly, these particles might be the micelles formed by the Tween or Span molecules released into the aqueous phase. The concept of using HP*β*CD as emulsifying agents in emulsion systems has been proven by several authors. HP*β*CD can stabilize emulsion systems by complexation of fatty acid residues of the oil phase and by reducing interfacial tension. Previously Klang et al. found that the excess of lecithin aggregates in the bulk water phase was caused by HP*β*CD molecules which were inserted into the interfacial film of the oil in water droplets [28, 29]. It is likely that the HP*β*CD may be incorporated with fatty acid residues of the oil phase. Secondly, HP*β*CD solubilizes the lutein and forms these complexes in the aqueous phase. These particles might be the noncovalent complex of lutein-HP*β*CD. In both cases, HP*β*CD can act as a “transporter” and enhances the leave of the lutein from the oil core into the cornea through complex formation. Therefore the lutein had more accumulation in cornea as compared to the HP*β*CD free vehicles. However, the exact composition of the population in the TEM remains to be investigated. Finally the addition of HP*β*CD represents an efficient way to promote the permeation of lutein without affecting the corneal barrier function.

**Figure 4:**
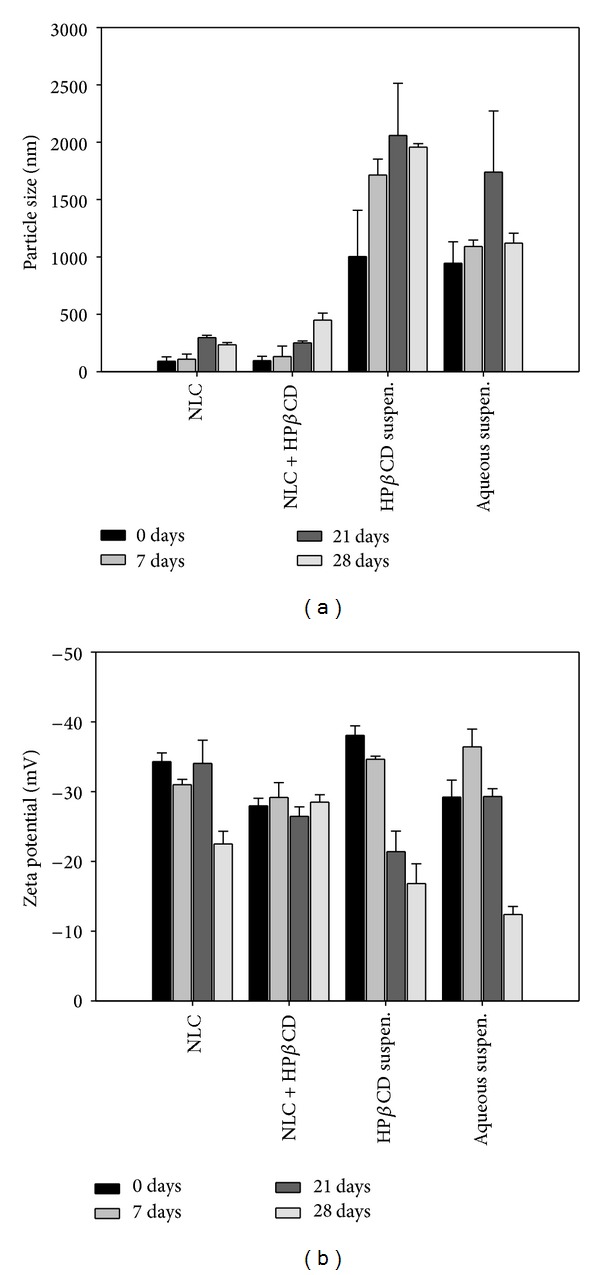
Size stability and zeta potential of lutein loaded vehicles after one month storage, *n* = 3.

The entrapment efficiencies for NLC and NLC + HP*β*CD are found to be 59% and 68%, respectively. The addition of HP*β*CD in NLC could enhance the encapsulation of lutein since the cave of HP*β*CD provided extraordinary space to accommodate lutein. The amount of corneal lutein accumulation using the NLC + HP*β*CD is found to be significantly higher than that of NLC. There are four factors, namely, enhancement of lutein solubility, large surface of nanoparticles, occlusive effect, and enhancer effect that contributed to the increased corneal delivery by NLC. The first reason is that lutein is hydrophobic and can be readily dissolved in NLC. This causes higher drug loading in the NLCs which in turn increases the concentration gradient towards the cornea. Secondly, the NLCs had more corneal lutein accumulation because the large surfaces ensure close contact and better adhesion on the cornea to deliver the lutein. However, the transport of intact nanoparticles into the cornea is difficult due to the barrier effects of the corneal epithelium [30]. Thirdly, the NLCs form films of densely packed spheres on the surface of the cornea which exert an occlusive effect by increasing corneal hydration [13]. This is the fact for increased corneal activity due to occlusive effect. Finally, the enhancer effect that increases corneal delivery by NLC might be due to the surfactants in the formulation. Surfactants which can loosen or fluidize the lipid bilayers on the corneal epithelium can act as permeation enhancers [31]. Formulation stability during storage is important for formulation development [32]. The effect of storage duration on the size and zeta potential of lutein-loaded vehicles was studied at the room temperature for four weeks. As indicated in Figure 4, NLCs could maintain their initial size and zeta potential during 4 weeks of the study period. NLCs with 2% HP*β*CD could maintain their initial size for 3 weeks. There are two possible locations that HP*β*CD molecule may occupy in the NLC vehicles. Since HP*β*CD can form the complex with fatty acid residues of the oil phase, HP*β*CD may exist in the oil/water interfaces. Another possibility is that HP*β*CD evenly distributes in the aqueous phase. The partition coefficients of HP*β*CD in interfaces and water determine its location. The exact location of HP*β*CD in the NLC formulation merits further investigation. However, the addition of HP*β*CD in the lipid nanoparticles can promote the accumulation of lutein in the ex vivo cornea model. HP-*β*CD could enhance the viability of corneal cells in the drug-free or lutein loaded NLCs.

Significant fluctuation of size distribution was observed for the aqueous and HP*β*CD suspensions during the storage. The size of the CD suspension and aqueous suspension was around 1000–2000 nm which was significantly larger than those of NLC vehicles. Absence of surfactants in these suspension accounted for the instability and large lipid colloids. Chemical transformation of solid lipids during storage is reported to change the structure of lipid core and release the drug [13]. The increase in particle size is a good indicator of instability since the aggregation and sedimentation is easy for these large particles. The zeta potentials for the four vehicles are found to be around −30 mV which could maintain the particles in suspension by repelling each other. The decrease in zeta potential was observed in the samples of NLC, aqueous suspension, and CD suspension during the 28-day storage. No lutein precipitation in the vehicles of NLC and NLC + HP*β*CD was observed during the storage at 25°C for 28 days. The developed NLCs and NLC + HP*β*CD are found to be stable when stored below 25°C for 4 weeks. NLCs also avoid drug expulsion caused by crystallization or lipid transformation of solid lipids nanoparticles upon cooling or storage [33]. The lutein distribution profile in the porcine cornea is evidenced by confocal laser scanning microscopy (CLSM).

### 3.3. Confocal Observations and Histological Examination of Porcine Cornea

**Figure 5:**
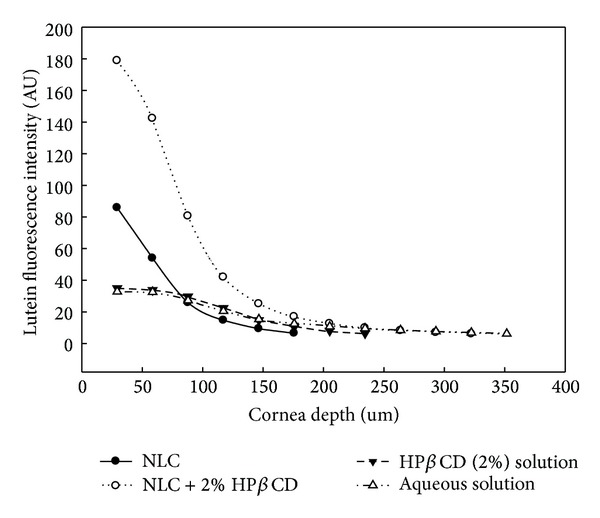
Lutein distribution in porcine cornea after 24 h treatment observed by using confocal laser scanning microscopy. Lutein fluorescence emitted at 515 nm was recorded when excited at a wavelength of 488 nm by means of an argon laser. The sample was scanned from the tissue surface (0 *μ*m) to a depth of 352 *μ*m at a 29.3 *μ*m interval.

CLSM provides the localization and permeation profile of fluorescent compounds in the transparent cornea without embedding procedures. When lutein is excited by 488 nm laser it emits light of wavelength 515 nm. This distribution of lutein in the cornea for NLC, NLC + HP*β*CD, HP*β*CD suspension and aqueous suspension is indicated in Figure 5. The lutein located at various corneal depths (from 0 to 352 *μ*m) was examined using CLSM. A depth of 0 *μ*m represents the corneal epithelium, whereas a depth of 352 *μ*m represents the stroma layer. Lutein in NLC + HP*β*CD could penetrate the most depth of 176 *μ*m into the cornea and could have the most fluorescence as compared to other vehicles. Since the thickness of corneal epithelium is 54 *μ*m [34], NLC + HP*β*CD could enter the corneal stroma. The fluorescence intensities of lutein in the skin were ranked in the order of NLC + HP*β*CD > NLC > HP*β*CD suspension = aqueous suspension. These results are consistent with corneal lutein accumulation using 4 vehicles shown in Figure 2. CLSM provides the direct evidence of distribution of lutein in the porcine cornea by using lutein fluorescence. The fluorescence intensities of lutein in NLC and NLC + HP*β*CD at the epithelial layer are found to be 179 and 86 arbitrary units (AU), respectively. The vehicle of NLC + HP*β*CD exhibited the largest lutein accumulation which provided a driving force for deeper lutein penetration. Very slight lutein penetration from the HP*β*CD suspension and the aqueous suspension was observed at 29 *μ*m depth. In contrast, lutein could penetrate to the depth of 117 *μ*m in the corneal stroma with the help of NLC vehicle. The HP*β*CD or aqueous suspension only had the fluorescence intensity of 35 AU at the outermost epithelium. The distribution profiles made from the CLSM data for lutein within the cornea tissue are observed to be vehicle dependent. The evidence of confocal intensity indicated that the lutein accumulation by NLC + HP*β*CD in the epithelium was twice higher than that of NLC alone. Since epithelial penetration of lutein is the first step for corneal accumulation, therefore the improved corneal accumulation in the presence of HP*β*CD may be due to the increase of lutein partition in the corneal epithelium. The lutein accumulation was proved again when HP*β*CD combined with NLC as the ocular delivery vehicles. In fact, the corneal lutein accumulation is controlled by the parameters such as diffusivity coefficient, path length, and partition coefficient in the corneal tissue. The type of cyclodextrins and lutein payload in the NLCs impacted the partition coefficient and sequentially affected the lutein accumulation. The proposed mechanism of HP*β*CD combined with NLC in the enhanced lutein accumulation includes the following possibility. In the process of corneal permeation, the drug should be released from the vehicles followed by partition or absorption into the cornea [35]. The diffusion rate of drugs from the vehicle is essential for the corneal accumulation of drugs [36]. The lutein molecules encapsulated into the fat matrix of NLCs had limited diffusion mobility. In contrast, the lutein loaded in HP*β*CD can be easily released to increase the lutein accumulation rate. Moreover, CD can extract cholesterol, phospholipids, and proteins from the cornea and can reduce the barrier effect of the corneal epithelium [15]. Finally, additional contact surfaces provided by CD might ensure more interact with corneal tissues [20].

### 3.4. Safety Evaluation of NLCs

**Figure 6:**
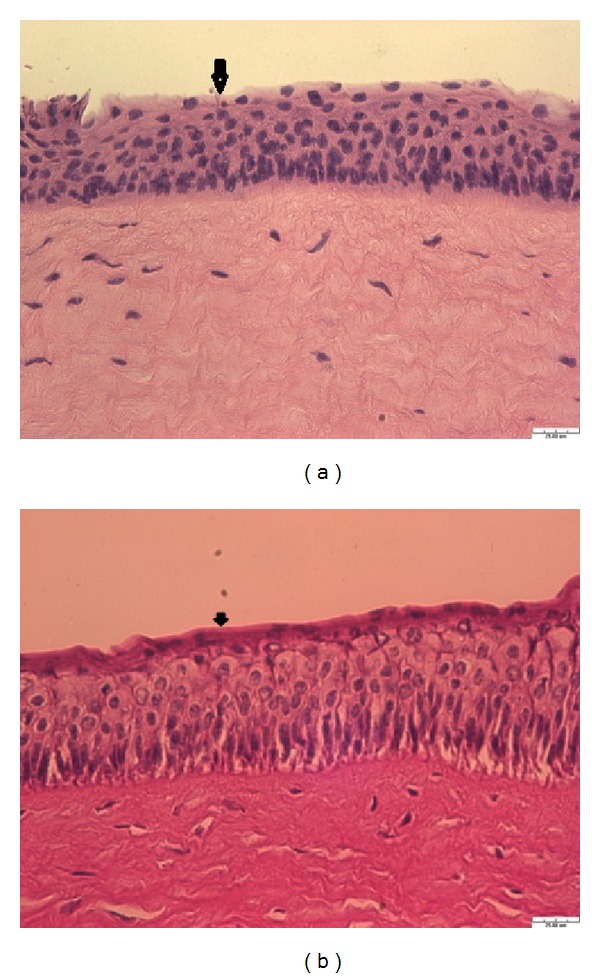
Micrographs of HE staining after 24 h treatment with PBS (a) and NLC + 2% HP*β*CD (b). Arrow indicates the epithelium. Scale bar represents 25 *μ*m. Porcine cornea after 24 h treatment was fixed, stained, and observed using light microscopy.

The histological examination of the porcine cornea by the NLC + HP*β*CD was analyzed using Hematoxylin and eosin (HE) staining in order to understand the impact of vehicles on the ocular tissue. Porcine cornea was treated with NLC + HP*β*CD and control (PBS) cornea for 24 hours before the HE staining. Micrographs of control and NLC + HP*β*CD treated corneas demonstrated normal histology of cornea as shown in Figures 6(a) and 6(b). Three layers of epithelium, Bowman membrane, and stroma could be clearly observed in the photos. Top of epithelial layer has some deposits of lutein and vehicles as compared to that of PBS treated cornea. Some puffy cells in the boundary between epithelium and Bowman membrane were found which might contribute to the enhanced effect of lutein delivery using the NLC + HP*β*CD vehicle. Cornea represents a five-layer barrier consisting of lipophilic epithelium (~50 *μ*m), hydrophilic stroma (~450 *μ*m), and lipophilic endothelium (monolayer) as well as Bowman membrane between epithelium and stroma and Descemet's membrane (between stroma and endothelium). Among the layers of cornea, the epithelium and endothelium are considered as lipophilic layers and the stroma is an aqueous layer. The epithelium contributes to resistance to hydrophilic drugs across the cornea [13]. The loose morphology of the epithelium treated by NLC + HP*β*CD (Figure 6) might act as the channel for the lutein accumulation and delivery into the cornea.

**Figure 7:**
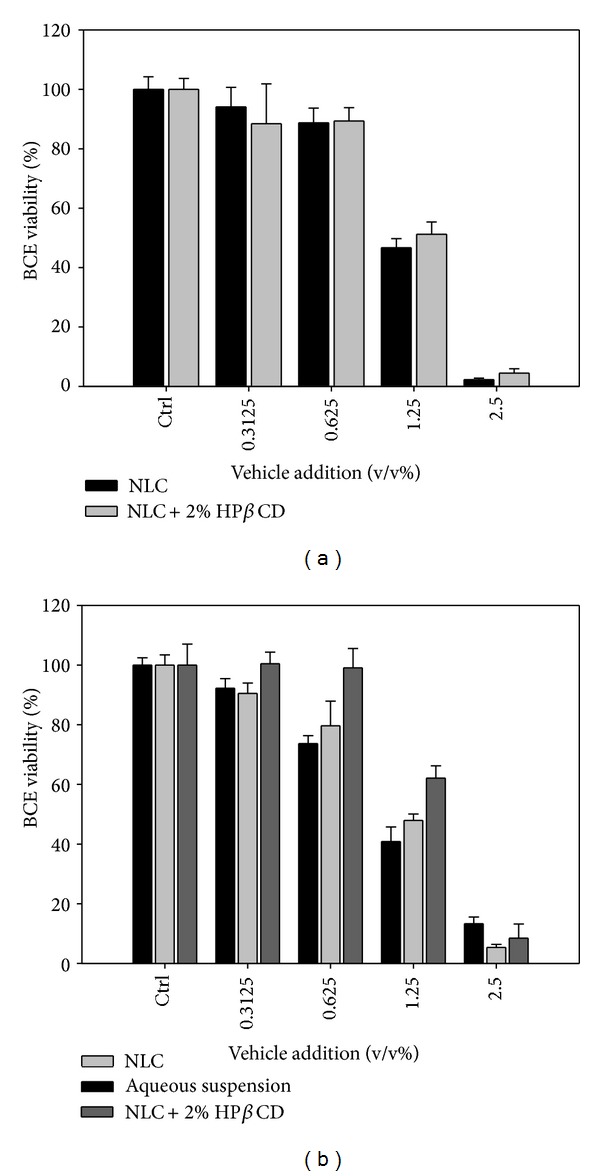
Cytotoxic effect of vehicles on bovine cornea epithelium cells: (a) vehicle only and (b) lutein loaded vehicles.

In order to evaluate the formula safety, the bare vehicles and drug loaded vehicles containing 2000 *μ*g g^−1^ lutein were used for the cytotoxicity experiments in BCE cells. The dilution range of vehicles is 0.2~2.5% using DMEM as the diluent. Similarly cell viability was observed for the bare and lutein-loaded vehicles as indicated in Figures 7(a) and 7(b). Lutein payload would not increase the toxicity as compared to the bare vehicles. The viability values for BCE cells at the dose of 1.25% of bare NLC + HP*β*CD and NLC were found to be 51.2% and 36.7%, respectively. Cytotoxic effects on BCE cells were alleviated when HP*β*CD added into the NLC in the drug-free or lutein loaded conditions. It has been noted that the toxicity of drug and carriers is decreased when CD incorporated in the formulation. In a previous study, remarkable cytotoxic effects on murine macrophage were found at 0.1% addition of free lipid nanoparticles [37]. The nanoparticles formulated with the stearic acid were cytotoxic at the 1% concentration for mouse J774 macrophages, 3T3 fibroblasts, and human HaCaT keratinocytes [38]. Very limited information is available for the toxicity effects of lipid nanoparticles on ocular cell lines. The results of the cytotoxicity test performed herein on BCE cells point to the importance of the formula ingredients used to prepare the lipid vehicles. Our data demonstrated that HP*β*CD in the NLC composition could increase the viability of BCE cells (Figure 7).

The physiological cornea condition is significantly different from the ex vivo experiments. However, several research groups choose similar conditions to evaluate the ex vivo drug delivery. For example, the effect of cyclodextrin on corneal permeability of riboflavin is studied in vitro using Franz diffusion cells with a 3-hour period. The steady-state flux and permeability in bovine cornea can be calculated by analyzing the aliquots taken from the receptor chamber every 30 min [24]. Additionally, the permeability coefficient of sodium fluorescein across fresh sclera is calculated by using a 4-hour in vitro diffusion apparatus [39, 40]. These ex vivo results are comparable as they are obtained under similar condition. However, the animal test is necessary for the further evaluation of the developed formulation.

In conclusion, lutein is a hydrophobic antioxidant associated with the macular degeneration. This study demonstrated the lutein localization in the porcine cornea by using the novel nanocarriers. However, lutein could not penetrate the whole porcine cornea under the tested conditions. We have explored the combinatory effect of CD and NLC on the corneal partition, encapsulation efficacy, stability, and distribution of lutein. The corneal accumulation and partition of lutein are improved by the developed vehicle. Also HP*β*CD enhanced the viability of corneal cells in the drug-free or lutein loaded NLCs. The permeation enhancement and nanoscaled properties of HP*β*CD combined with NLCs contributed to the enhanced accumulation of lutein in the cornea.

## Figures and Tables

**Table 1:** Effect of cyclodextrins (CD) on corneal lutein accumulation in combination with NLC∗.

Vehicles	Accumulation rate (*μ*g/g/hr)	Partition coefficient (10^−3^)	Enhance ratio
NLC	10.6 ± 0.4	21.25 ± 0.8	1
NLC + *α*CD	12.1 ± 3	24.20 ± 6.2	1.14
NLC + *β*CD	16.2 ± 9	32.40 ± 1.5	1.52
NLC + HE*β*CD	16.2 ± 3.5	32.45 ± 7.1	1.53
NLC + HP*β*CD	19.8 ± 0.2	39.80 ± 0.6	1.80

*The CD and lutein content in the tested vehicles is 0.5% and 2000 *μ*g/g, respectively.

**Table 2:** Summary of corneal lutein delivery using CD combined with NLCs.

Vehicles	Lutein content (*μ*g/g)	Accumulation rate (*μ*g/g/hr)	Partition coefficient (10^−3^)	Enhance ratio
NLC	2000	10.6 ± 0.4	21.25 ± 0.8	1
NLC + 0.5% HP*β*CD	2000	19.8 ± 0.2	39.8 ± 0.55	1.80
NLC + 1% HP*β*CD	2000	23 ± 1.8	46 ± 3.7	2.17
NLC + 2% HP*β*CD	2000	52.3 ± 5	104.6 ± 9	4.92
NLC + 3% HP*β*CD	2000	27 ± 1.6	54.2 ± 3.2	2.55
NLC + 2% HP*β*CD	4000	142.8 ± 3.0	142.8 ± 5.7	13.44

**Table 3:** Composition and characteristics of vehicles loaded with lutein*.

Composition (%)	Vehicles			
	NLC	NLC + HP*β*CD	HP*β*CD suspension	Aqueous suspension
Lutein/Transcutol	0.2/19	0.2/19	0.2/19	0.2/19
Corn oil	0.8	0.8	0.8	0.8
Capric acid	1	1	—	—
Tween 80	3	3	—	—
Span 60	2	2	—	—
HP*β*CD	—	2	2	—
Water	74	72	78	80
Property				
Mean (nm)	229.8 ± 65	336.8 ± 43	1003 ± 403	945 ± 186
Zeta potential (mV)	−34.3 ± 0.2	−28.0 ± 0.5	−38.1 ± 1.2	−29.2 ± 2.2
Entrapment efficiency (%)	59.0 ± 1.5	68.1 ± 3.3	0.19 ± 0.01	0.06 ± 0.02
Partition coefficient (10^−3^)	21.25 ± 0.8	104.6 ± 0.9	6.6 ± 2.5	0.3 ± 0.05

*The lutein content in all vehicles is 2000 *μ*g/g.
